# Crystal structure analysis of van der Waals layered phospho­rus chalcogenide CuVP_2_S_6_

**DOI:** 10.1107/S2052520625008820

**Published:** 2025-11-12

**Authors:** Nur Farhana Hasmuni, Daisuke Urushihara, Ryura Hayashi, Ryo Sato, Koichiro Fukuda, Mohd Zamri Mohd Yusop, Toru Asaka

**Affiliations:** a Pusat Pengurusan Makmal Universiti (PPMU), University Industry Research Laboratory (UIRL), Universiti Teknologi Malaysia, UTM Johor Bahru, Johor, 81310, Malaysia; b Division of Advanced Ceramics Nagoya Institute of Technology Gokiso-cho Nagoya Aichi466-8555 Japan; c Department of Materials, Manufacturing and Industrial Engineering, Faculty of Mechanical Engineering Universiti Teknologi Malaysia 81310 UTM Johor Bahru Johor Malaysia; University of Antwerp, Belgium

**Keywords:** van der Waals layered compound, transmission electron microscopy, single-crystal X-ray diffraction

## Abstract

The crystal structure of CuVP_2_S_6_ is investigated by transmission electron microscopy (TEM) and single-crystal X-ray diffraction. Cu, V and dimerized P_2_ are each octahedrally coordinated by S atoms. Each metal ion species forms a triangular lattice, which is interdigitated to form a two-dimensional sheet. Each sheet forms a layered structure with van der Waals gaps between them. The crystal has a rotational twin structure, which is manifested in the form of stacking disorder. Refined structure analysis reveals characteristic rotational distortion of the octahedra. The existence of Cu sites protruding into the van der Waals gap is directly shown by high-resolution scanning TEM.

## Abstract 2

Two-dimensional van der Waals layered compound CuVP_2_S_6_ is studied by transmission electron microscopy and single-crystal X-ray diffraction. Rotational microtwin structures, which may be typical and characteristic of two-dimensional van der Waals compounds, are observed.

## 1. Introduction

In recent years, two-dimensional materials with van der Waals (vdW) gaps have attracted increasing interest due to their potential for device applications and scientific novelty (Radisavljevic *et al.*, 2011 ▸; Xu *et al.*, 2017 ▸; Wang *et al.*, 2019 ▸; Jia *et al.*, 2022 ▸; Chen *et al.*, 2022 ▸; Liu *et al.*, 2016 ▸; Duan *et al.*, 2022 ▸; Zhao *et al.*, 2020 ▸; Bian *et al.*, 2022 ▸; Chen *et al.*, 2024 ▸; Cao *et al.*, 2012 ▸; Chen *et al.*, 2013 ▸; Desai *et al.*, 2016 ▸; Huang *et al.*, 2017 ▸; Gong *et al.*, 2017 ▸). These materials enable the creation of novel heterostructures and devices with tunable electronic, optical, magnetic and energy-related functionalities (Chen *et al.*, 2024 ▸; Zhang *et al.*, 2022 ▸; Burch *et al.*, 2018 ▸; Zhang *et al.*, 2025 ▸). Among them, metal phospho­rous trichalcogenides, represented by the chemical formula *M*P*X*_3_ (*M*: divalent metal ion, *X*: S or Se), have been intensively studied as two-dimensional magnetic materials because they exhibit magnetism due to transition metal ions occupying the *M* site (Hahn & Klingen, 1965 ▸; Duan *et al.*, 2022 ▸; Chittari *et al.*, 2016 ▸; Peng & Tong, 2025 ▸). In addition, *M*′*M*′′P_2_*X*_6_ (*M*′: Cu^+^ or Ag^+^, *M*′′: In^3+^, Cr^3+^, V^3+^, Sc^3+^*etc*.), derived from *M*P*X*_3_, have been successfully prepared (Colombet *et al.*, 1982 ▸; Lee *et al.*, 1986 ▸; Ouili *et al.*, 1987 ▸; Durand *et al.*, 1990 ▸; Maisonneuve *et al.*, 1995 ▸; Colombet *et al.*, 1983 ▸; Lee *et al.*, 1988 ▸). Such compounds have attracted attention as ferroelectric and piezoelectric materials, because ferroelectricity due to a polarization by the large atomic displacement of *M*′ ions toward the outside of the layer, have been observed in CuInP_2_S_6_ (Maisonneuve *et al.*, 1997 ▸; Belianinov *et al.*, 2015 ▸; Liu *et al.*, 2016 ▸; Balke *et al.*, 2018 ▸; Zhou *et al.*, 2021 ▸; Zhao *et al.*, 2020 ▸; You *et al.*, 2019 ▸; Jia *et al.*, 2022 ▸). Furthermore, interestingly, a giant negative piezoelectric effect has been also observed in CuInP_2_S_6_. In addition, when the *M*′′ ion is a transition metal, this *M*′*M*′′P_2_*X*_6_ has magnetic properties and the possibility of a magnetic dielectric has been shown. In fact, multiferroic properties have been experimentally observed in CuCrP_2_S_6_ (Lai *et al.*, 2019 ▸; Park *et al.*, 2022 ▸; Wang *et al.*, 2023 ▸).

In Cu*M*′′P_2_S_6_, CuS_6_, *M*′′S_6_ and P_2_S_6_ octahedra share edges with each other to form a two-dimensional sheet (Cajipe *et al.*, 1996 ▸). However, most of the Cu ions are located away from the center of the octahedron, close to the face of the octahedron facing the vdW gap and most of the Cu ions are considered to be three-coordinated with S ions. Cu ions, *M*′′ ions and P_2_ dimers can be considered to form triangular lattices. When such two-dimensional sheets are stacked in the c* direction across the vdW gap, in CuInP_2_S_6_ and CuCrP_2_S_6_, the next sheet that is mirror-symmetric with respect to the plane through the *M*′′ or Cu sites perpendicular to the sheet, is stacked by shifting 1/3 of the unit cell, *i.e* [1/3, 0, 0], along the *a* axis (Maisonneuve *et al.*, 1995 ▸; Cajipe *et al.*, 1996 ▸). In other words, these sheets are built from edge-sharing octahedra and exhibit unique stacking and symmetry features, including *c*-glide symmetry (Zhou *et al.*, 2024 ▸; Balke *et al.*, 2018 ▸). Consequently, the crystal structures of CuInP_2_S_6_ and CuCrP_2_S_6_ at room temperature are monoclinic *Cc* (Maisonneuve *et al.*, 1995 ▸) and *C*2/*c* (Colombet *et al.*, 1982 ▸; Maisonneuve *et al.*, 1995 ▸), respectively, and contain two sheets in the unit cell. Combining their symmetries with the ordered arrangement of Cu atoms with atomic coordinate freedom in the c* direction within the sheet, CuInP_2_S_6_ and CuCrP_2_S_6_ exhibit polar (Maisonneuve *et al.*, 1995 ▸; Zhou *et al.*, 2020 ▸) and antipolar (Maisonneuve *et al.*, 1993 ▸) structures, respectively. In contrast, it has been reported that, in CuVP_2_S_6_, the sheets are stacked with only a 1/3 shift along the *a* axis, *i.e.* [1/3, 0, 0] and there is no *c*-glide symmetry between adjacent sheets. As a result, the monoclinic *C*2 (unit-cell parameters *a* = 5.9462 Å, *b* = 10.2990 Å, *c* = 6.6870 Å, β = 107.250°) is formed (Durand *et al.*, 1990 ▸). Here, the unit-cell parameter *c* is half that of CuInP_2_S_6_ and CuCrP_2_S_6_, *i.e.* there is only one sheet in the unit cell.

As mentioned above, the electric polarization of the Cu*M*′′P_2_S_6_ system is believed to be due to the atomic displacement of Cu (or the occupancy of Cu atomic sites) and it is roughly oriented along the c* axis. Ferroelectricity has not yet been reported in CuVP_2_S_6_. However, since it has the same Cu^+^ ions as CuInP_2_S_6_ and CuCrP_2_S_6_ (and even has Cu sites protruding into the vdW gap), it is natural to expect (anti)ferroelectricity to be exhibited in CuVP_2_S_6_. However, in the space group *C*2, the polarization direction is strictly determined to be the *b* axis direction and electric polarization in the c* direction is not allowed. Duan *et al.* have predicted a correlation between electric polarization and magnetic order in nanosheets of CuVP_2_S_6_ consisting of several layers, based on first-principles calculations and theoretical models (Duan *et al.*, 2022 ▸). However, their structural models for CuVP_2_S_6_ appear to be isomorphous with that of CuInP_2_S_6_ and differ from the structural model (space group *C*2) experimentally analyzed as mentioned above.

In the structural analysis by Durand *et al.* mentioned above (Durand *et al.*, 1990 ▸), the Cu site located near the center of the CuS_6_ octahedron, which is labeled as Cu1 in the paper, is represented as a split-atom model with no characteristics of coordination environment and the details of the structure seem unreasonable. Based on this and the comparison with other Cu*M*′′P_2_S_6_ mentioned above, the crystal structure of CuVP_2_S_6_ deserves to be re-examined. In addition, the existence of the Cu site protruding into the vdW gap, which is referred to as the Cu3 site in Durand *et al.* (1990 ▸), has not been directly observed, even though it is important not only for ferroelectricity such as polarization switching but also for ionic conductors (Guo *et al.*, 2024 ▸; Zhou *et al.*, 2023 ▸). Therefore, we synthesized crystal samples of CuVP_2_S_6_ and re-examined the crystal structure by electron diffraction and single-crystal X-ray diffraction techniques and direct imaging using high-resolution scanning transmission electron microscope (STEM).

## 2. Experimental

Single crystalline bulk CuVP_2_S_6_ was synthesized using a dry mixing method followed by solid-state growth. The elemental of copper powder (Cu, 97.0%), vanadium metal powder (V, 99.5%), phospho­rus powder (P, 98.0%) and sulfur powder (S, 99.0%), were combined in precise stoichiometric proportion, weighed and subsequently vacuum-sealed in quartz tubes. These sealed tubes were then placed vertically in a furnace. The tubes were slowly heated under vacuum to a temperature of 500°C, and were maintained at 500°C for 11 days. Following this period, the tubes were allowed to cool naturally to ambient temperature. This process resulted in the formation of aggregated polycrystals as well as thin, plate-like single crystals.

The TEM specimens were prepared by ion milling. The single crystals and the aggregated polycrystals were embedded in resin, cut into thin plates and then thinned by Ar^+^ ion beam irradiation. Selected-area electron diffraction (SAED) and scanning transmission electron microscopy (STEM) observations were carried out using transmission electron microscope JEM-ARM200F (JEOL) at 200 kV. Simulations of high-resolution STEM images were performed using *xHREM* (HREM Research Inc.) (Ishizuka & Uyeda, 1977 ▸), based on a multislice computational method.

X-ray diffraction data were collected using a single-crystal X-ray diffractometer (D8 VENTURE, Bruker) equipped with a Mo *K*α X-ray source (50 kV, 1 mA). Unit-cell parameters were determined using the *SAINT* (Bruker, 2015 ▸) program and multiscan absorption correction was performed using the *SADABS* (Bruker, 2015 ▸) program. The initial structure model was calculated using the *Superflip* (Palatinus & Chapuis, 2007 ▸) program, which is based on a charge-flipping algorithm. The crystal structure analysis was performed using the *JANA2006* (Petříček *et al.*, 2014 ▸) program package and the crystal structure was visualized using the *VESTA* (Momma & Izumi, 2011 ▸) program.

The crystal data and structural parameters are shown in Tables 1 ▸ and 2 ▸, respectively.

## 3. Results and discussion

**Figure 1:**
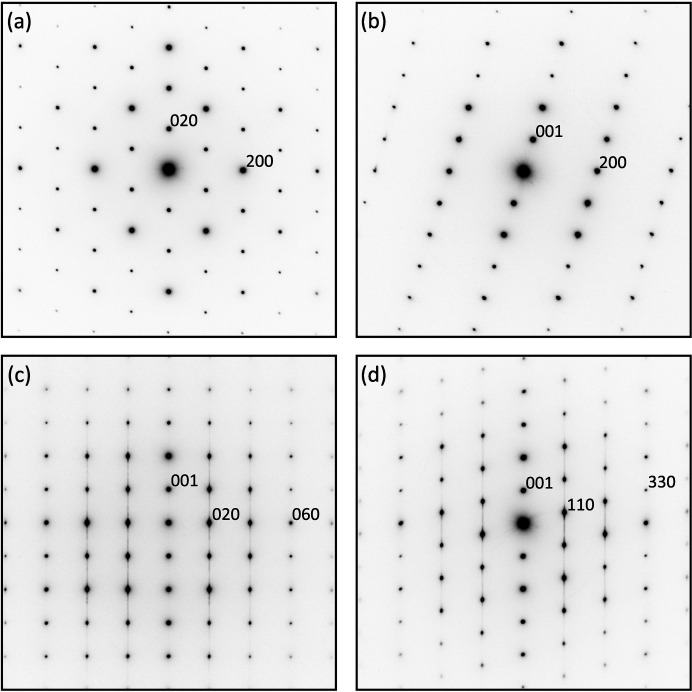
Sets of electron-diffraction patterns of CuVP_2_S_6_ showing zones of axes (*a*) [001], (*b*) [010], (*c*) [100] and (*d*) [110].

Fig. 1 ▸ shows typical selected area electron diffraction patterns (SAED) of CuVP_2_S_6_. As can be seen, almost all reflections are sharp and spot-like, but weak diffuse streaks along c* axis are observed in the diffraction patterns for certain electron beam incidence directions, as seen in Figs. 1 ▸(*c*) and 1 ▸(*d*). The origin of these will be discussed later. From the SAED patterns obtained from various crystallographic orientations, the only systematic extinction found is *hkl*: *h* + *k* = 2*n* (*n*: integer). This is for a *C* base-centered lattice, and the possible space groups for this material are *C*2, *Cm* and *C*2/*m*.

**Figure 2:**
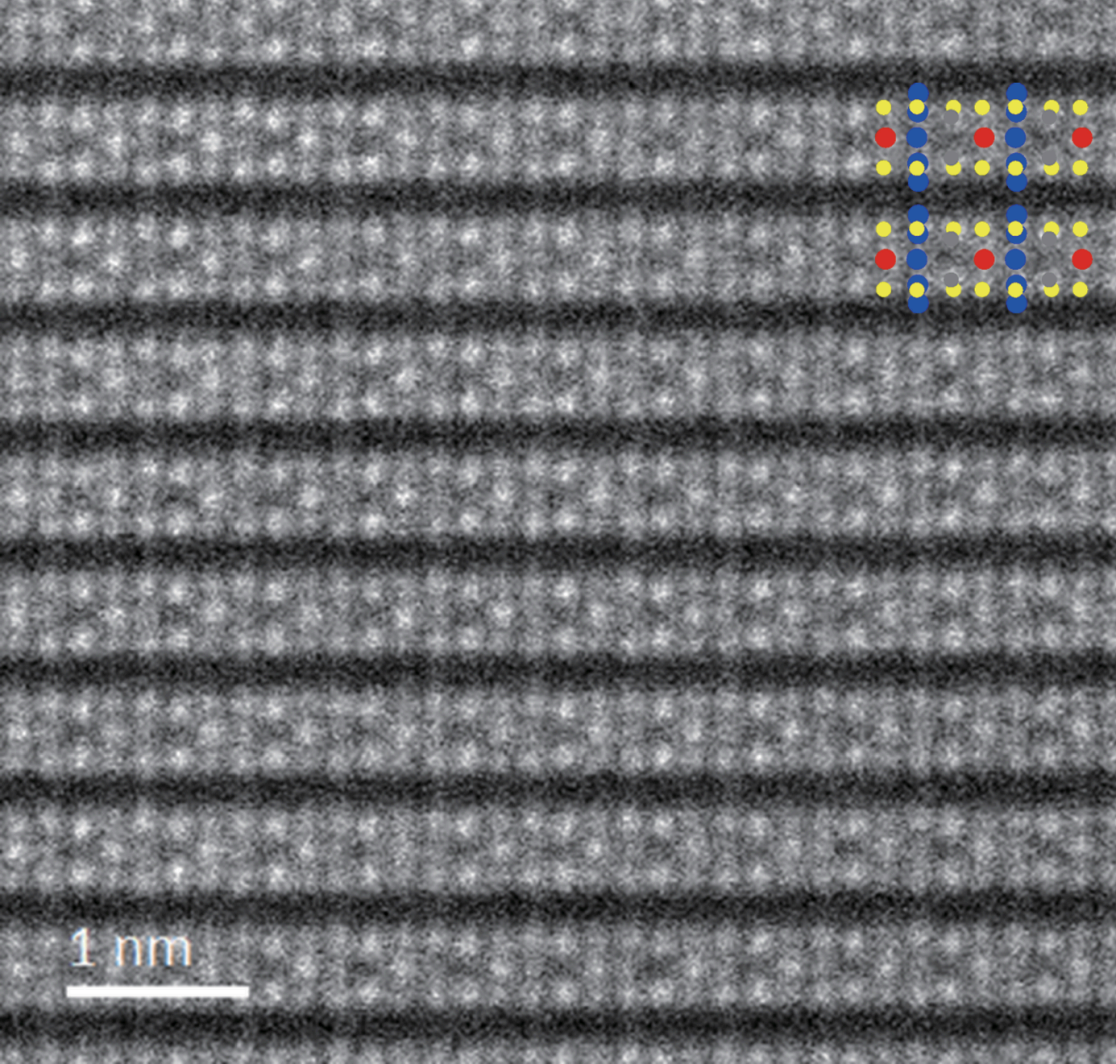
High-angle annular-dark-field (HAADF)-STEM image of CuVP_2_S_6_, taken along the [100] direction. The inset is the crystal structure model. Blue, red, gray and yellow circles denote Cu, V, P and S, respectively.

To verify which of the candidate space groups is most likely, we performed real-space observations of the atomic arrangement by high-angle annular dark-field (HAADF)-STEM. Fig. 2 ▸ shows a [100] zone axis HAADF-STEM image, corresponding to Fig. 1 ▸(*c*). Highly regularly aligned atomic columns are observed. The vertical direction of this image is parallel to the c* axis and the horizontal direction, *i.e.* the direction along the layers, is the *b* axis. Among the candidate space groups, *Cm* and *C*2/*m* have mirror symmetrical planes perpendicular to the *b* axis. Therefore, if this material has these space groups, it should be possible to find a mirror symmetry plane perpendicular to the layers, *i.e.* along the vertical direction of Fig. 2 ▸, in the HAADF-STEM image. However, no such mirror symmetry plane was found. Thus, the space group of this material was unambiguously determined to be *C*2.

**Figure 3:**
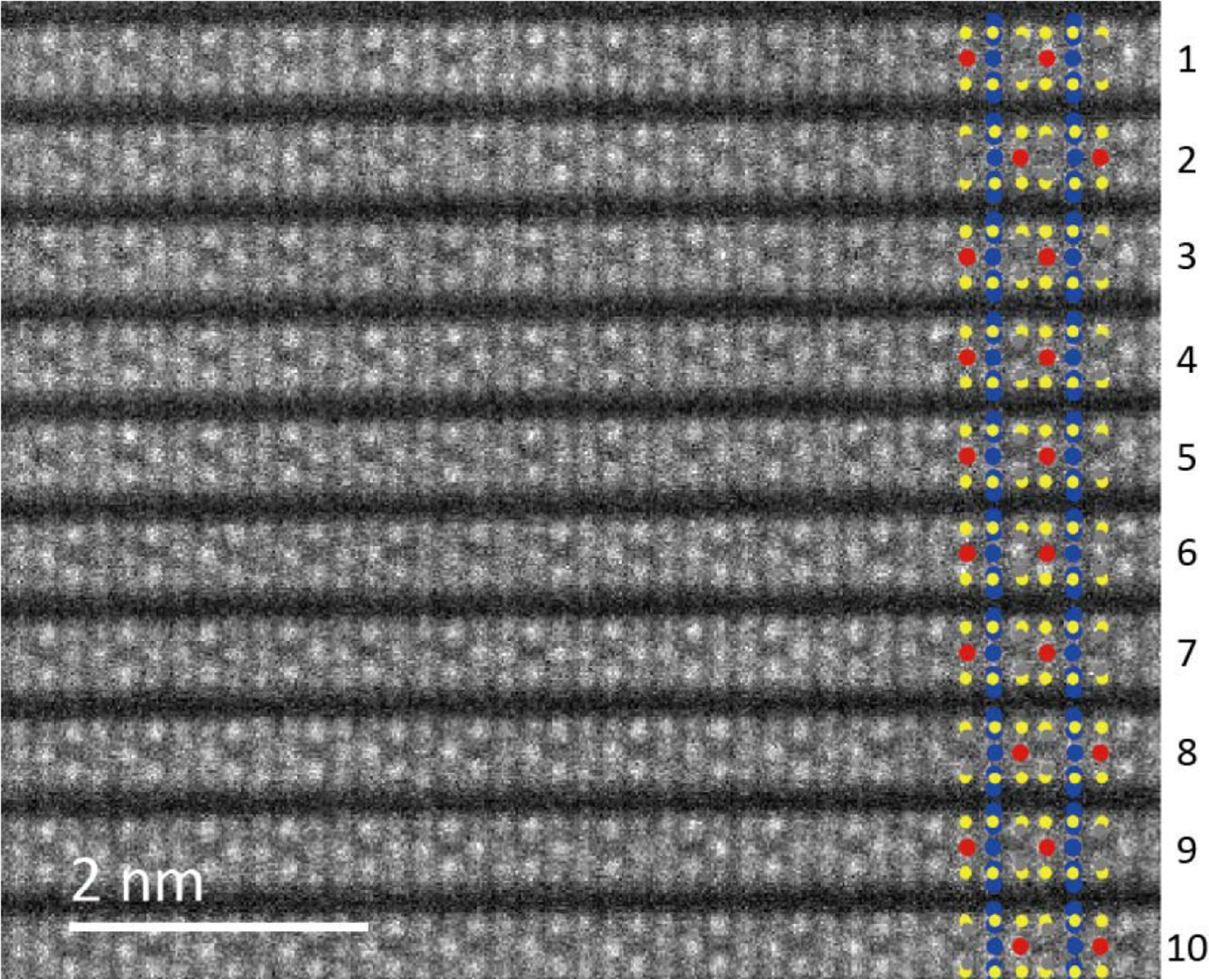
HAADF-STEM image of CuVP_2_S_6_, taken along the [100] direction. The inset is the crystal structure models, showing the atomic arrangement for each layer. The numbers on the right side of the figure are the layer indexes. The arrangement of metal atoms in layers 2, 8 and 10 is in the opposite order as seen along the layers.

Fig. 3 ▸ shows a HAADF-STEM image of a different region of the crystal from which Fig. 2 ▸ was obtained. In this region, the atomic arrangement within each layer is also perfect, but in the stacking direction, there are several places along the layers where the atomic arrangement is reversed (see layers 2, 8 and 10). In these layers, the layers are rotated 180° around the c* axis, or mirror symmetry operations are performed with the Cu sites as the mirror plane. These are a kind of stacking fault and can be regarded as insertion of microtwins. Although such microtwin structure is characteristic of 2D vdW compounds, 180° rotation twin structure has not been observed in FePS_3_, a similar vdW compound (Murayama *et al.*, 2016 ▸). Such stacking faults cause diffuse streaks along c* in the SAED. Diffuse streaks connecting the diffraction spots are present in 02*l* and 04*l*, but not in 06*l*. The (030) spacing roughly corresponds to the spacing of the atomic columns perpendicular to the layers when viewed in this orientation, S-Cu-S, S-V-S, S-P-P-S, but if these atomic columns are considered to be identical, layers 2, 8 and 10 are also not considered to be in reverse order. In other words, there is no stacking disorder for this structural feature (atomic arrangements) and no diffuse streaks appear in the 06*l* reflections. Interestingly, even for layers with reversed atomic order, the Cu sites are always directly above and below the adjacent layers on either side.

**Figure 4:**
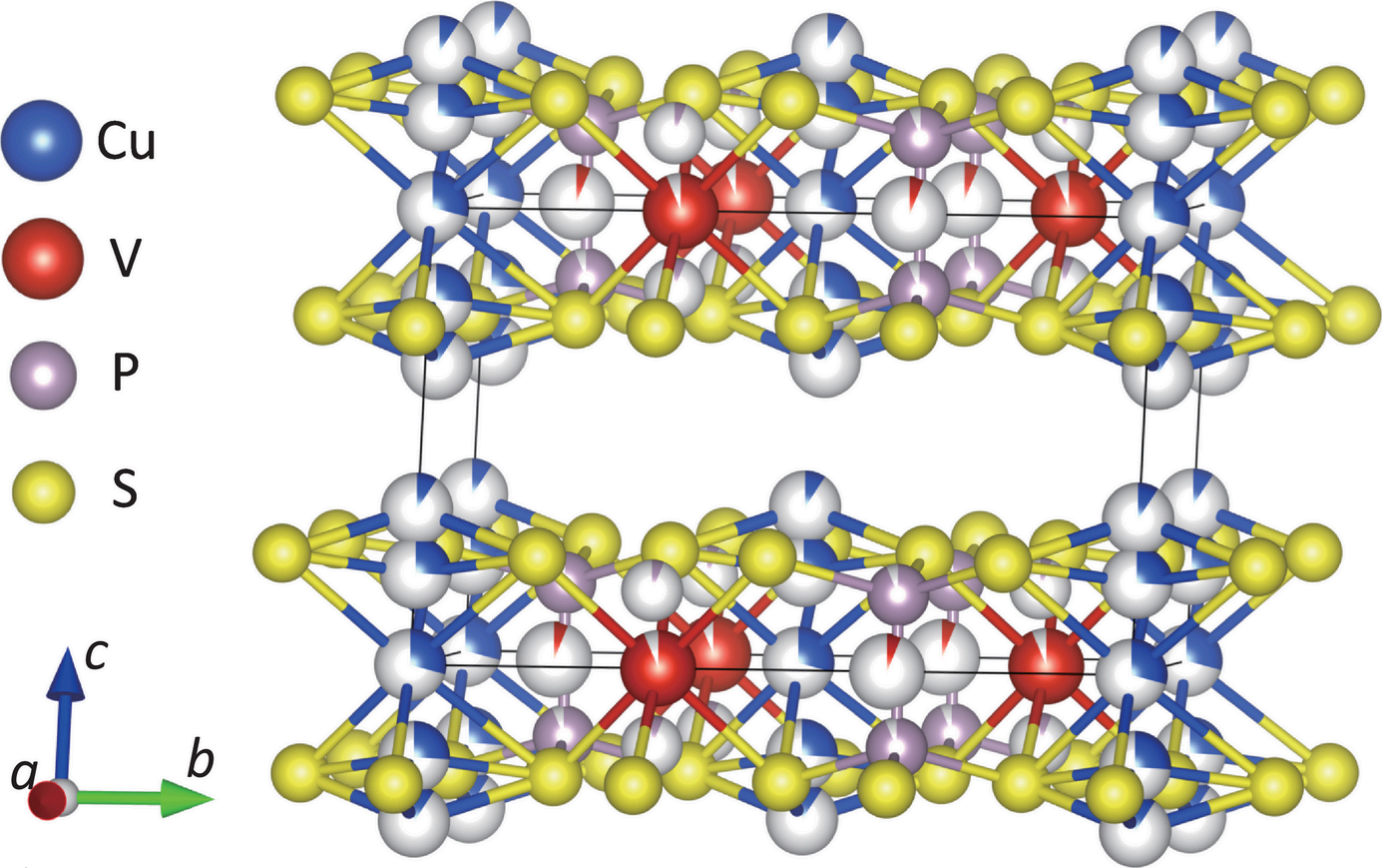
Crystal structure model of CuVP_2_S_6_. Blue, red, gray and yellow spheres denote Cu, V, P and S, respectively. The Cu site is represented by a split-atom model, while the partial occupancy of the V and P sites is due to the microtwin structure.

**Figure 5:**
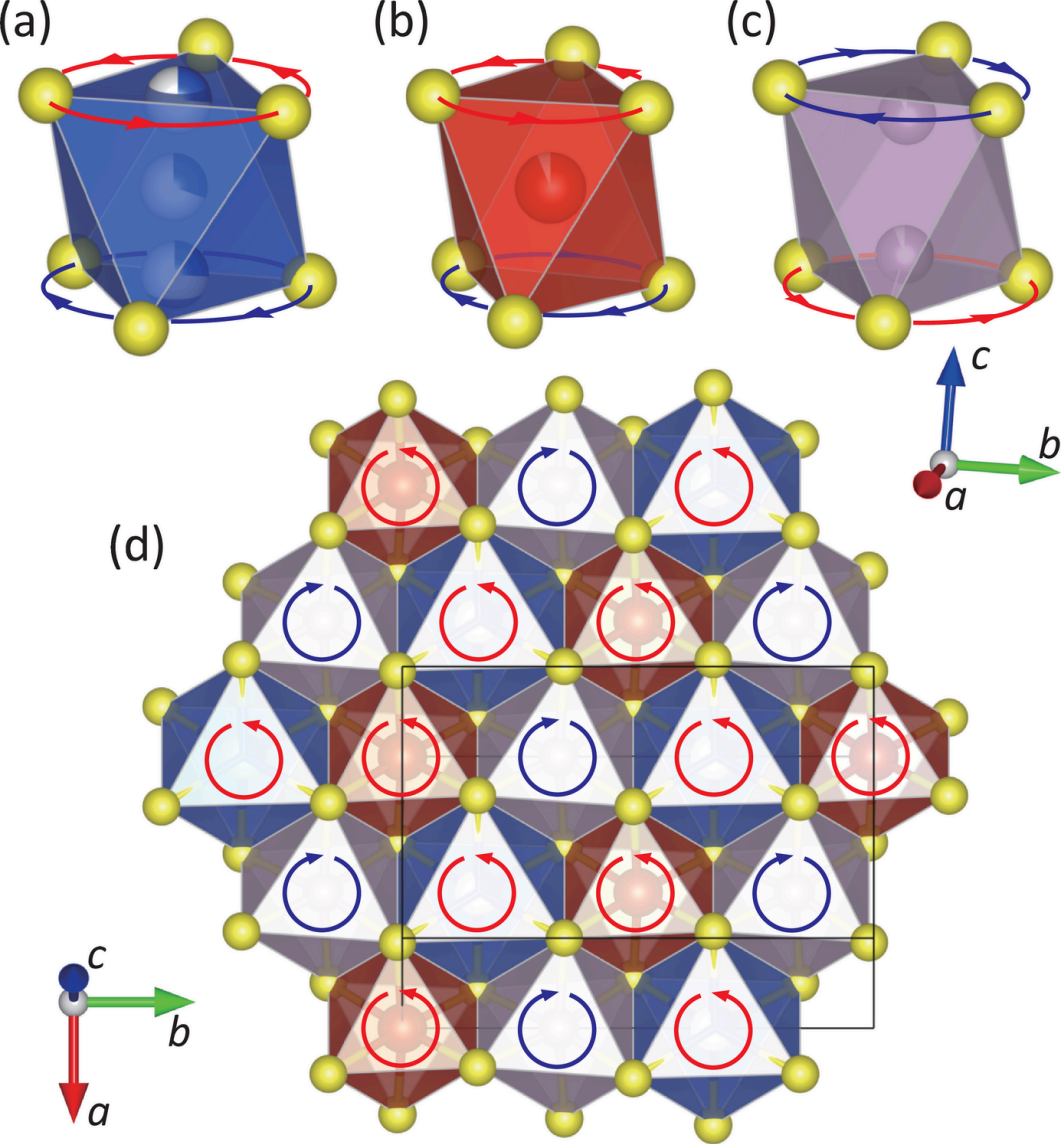
Partial structure models of the metal–sulfur octahedron of (*a*) CuS_6_, (*b*) VS_6_ and (*c*) P_2_S_6_. (*d*) The crystal structure model of a single two-dimensional sheet viewed from the c* axis. The representation color of each octahedron is the same as that of each atom. For the crystal axis compass, see the one on the right for (*a*)–(*c*) and the one on the lower left for (*d*). In all figures, arrows represent directions of rotational displacements of each basal plane.

The crystal structure was analyzed using single-crystal X-ray diffraction data with the space group *C*2. The obtained crystal structure model is shown in Fig. 4 ▸. First, in order to clarify the essential structural features, we will discuss the structure focusing only on the highly occupied sites of Cu, V and P, *i.e.* Cu1, Cu2, V1 and P1. In CuVP_2_S_6_, six S atoms coordinate to the respective metal ions, Cu, V and P_2_, forming coordination octahedra [Figs. 5 ▸(*a*)–5(*c*)]. These octahedra share edges within each sheet, forming a two-dimensional triangular lattice with respect to the metal ions. The respective metal ions also form triangular lattices, which are nested in an ordered arrangement [Fig. 5 ▸(*d*)]. Each sheet is stacked while shifting 1/3 along the *a* axis, *i.e.* [1/3, 0, 0]. In addition, a split-atom model represented by three Cu sites, where a Cu site is divided to Cu1, Cu2 and Cu3, was analytically considered to be appropriate. The above structural features are the same as those of the previously reported structure by Durand *et al.* (1990 ▸). Incidentally, the problem with the Durand *et al.* structural model mentioned above, the split of the Cu1 site, which cannot be explained well from a crystal chemistry perspective, was successfully avoided by placing Cu1 on a twofold axis, *i.e.* site symmetry 2. This approach reduced the reliability factor for the structural analysis sufficiently. When the model omitting V2 and P2 from this crystal structure model is superimposed on the HAADF-STEM image in Fig. 2 ▸, it can be seen that the atomic arrangements match well. On the other hand, the expression of partial occupancy of the V and P sites is not intrinsic feature of the crystal structure, but is thought to be due to the existence of the microtwin described above. In the STEM images, any antisite defect between the V and P sites within the layer has not been observed and the Cu sites are always directly above and below the adjacent layers. Therefore, the distribution of site occupancy to the V2 and P2 sites is assumed to be the result of the microtwin portion, as shown in Fig. 3 ▸. By the way, it is interesting to note that in this structural model, the opposing bases of each *M*S_6_ octahedron are rotated in opposite directions [Figs. 5 ▸(*a*)–5(*c*)]. Such rotational distortion features have also been found in two-dimensional transition metal compounds with vdW gaps, such as α-RuCl_3_ (Cao *et al.*, 2016 ▸; Banerjee *et al.*, 2016 ▸) and VI_3_ (Tian *et al.*, 2019 ▸). When viewed along the c* axis, the octahedra in these compounds are rotated in phase with CuS_6_ and VS_6_ and in antiphase with P_2_S_6_ [Fig. 5 ▸(*d*)].

**Figure 6:**
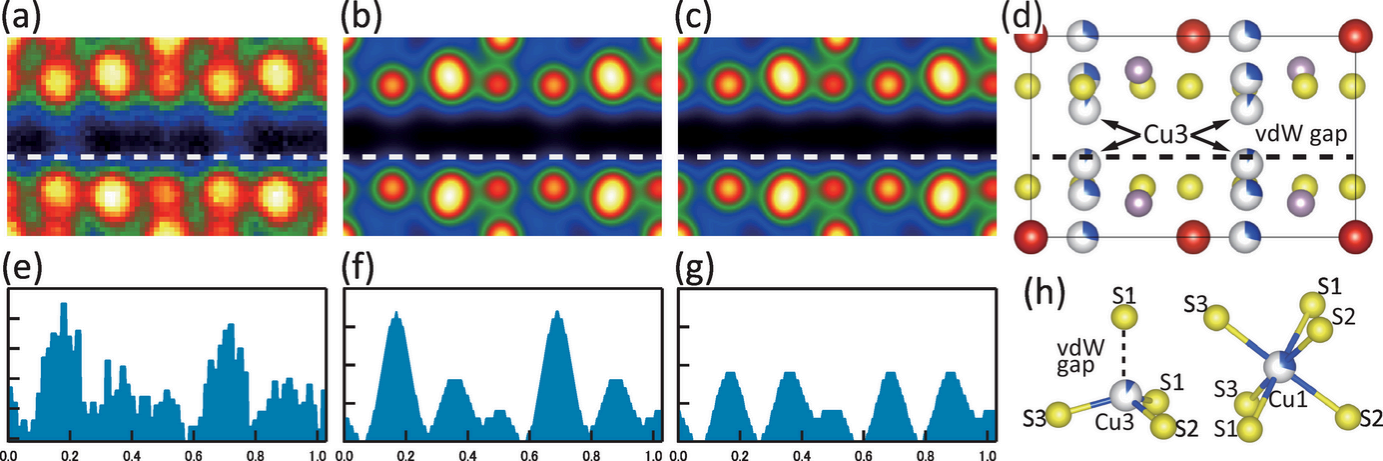
(*a*) HAADF-STEM image of the unit cell, obtained by segmenting the entire area of the image in Fig. 2 ▸ into unit cells, extracting them, superimposing and averaging. Simulation HAADF-STEM image of the unit cell for the crystal structure model (*b*) with and (*c*) without Cu3 atoms, respectively, projected along [100]. (*d*) Crystal structure model, viewed along [100]. (*e*)–(*g*) The intensity profiles on the white dashed lines in the HAADF-STEM and simulation images (*a*)–(*c*), respectively. Significant intensity is obviously observed at the Cu3 position in images (*a*) and (*b*). (*h*) Partial structure models around (left) Cu3 and (right) Cu1 atoms.

Finally, we discuss the Cu3 site that protrudes into the vdW gap. Although the Cu3 site is a site with a very low occupancy in the analysis, it is certainly present by analyzing the HAADF-STEM image. Fig. 6 ▸(*a*) shows the images of the unit cell extracted from the entire HAADF-STEM image of Fig. 2 ▸, which were then superimposed and averaged. Here, to aid in the visualization of the intensity profile, the origin of the unit cell has been shifted by (0, 0.3437, 0) from the structural analysis results shown above so that the V1 site is the origin. Fig. 6 ▸(*b*) shows a simulation of the HAADF-STEM image based on the single-crystal X-ray structural analysis results. Here, the field of view in Fig. 2 ▸ does not include layers rotated 180° about the c* axis corresponding to V2 and P2, so the simulation image was calculated assuming a structural model in which V1 and P1 have an occupancy of 1 and V2 and P2 do not exist, as shown in Fig. 6 ▸(*d*). In addition, to verify the existence of Cu3 sites, a simulation was also performed for a model without Cu3 sites. In this case, the Cu originally occupying the Cu3 sites was distributed according to the ratio of the occupancy rates of the Cu1 and Cu2 sites. The simulated image calculated in this way is shown in Fig. 6 ▸(*c*). Comparing the HAADF-STEM image in Fig. 6 ▸(*a*) with the simulated images in Figs. 6 ▸(*b*) and 6 ▸(*c*), they are almost identical, supporting the validity of the structural model obtained by single-crystal X-ray structure analysis. Figs. 6 ▸(*e*), 6 ▸(*f*) and 6 ▸(*g*) show the image intensity profiles on the white dashed lines in Figs. 6 ▸(*a*), 6 ▸(*b*) and 6 ▸(*c*), respectively. Fig. 6 ▸(*g*) shows four maxima, while Figs. 6 ▸(*e*) and 6 ▸(*f*) show maxima at locations corresponding to two Cu3 sites and it can be seen that the two images are in good agreement with each other. Therefore, even a small amount of Cu occupying the Cu3 site was clearly reflected in the image intensity of the HAADF-STEM image and this analysis directly proved the existence of Cu3 sites in this material.

Fig. 6 ▸(*h*) shows the relative positions of S ions adjacent to Cu3 and Cu1, respectively. As shown in Table 3 ▸, the interatomic distance between Cu3 and S1 across the vdW gap is 2.58 (2) Å, which is shorter than the two interatomic distances between Cu1 and S in the Cu1S_6_ octahedron, Cu1–S1 = 2.648 (2) Å and Cu1–S2 = 2.691 (7) Å and is almost the same as the interatomic distance between Cu1 and S3, 2.573 (6) Å. This suggests that there is a bond between Cu3 and S1 across the vdW gap. In other words, Cu3 would strengthen vdW coupling, while the crystal remains layered overall. In addition, Cu3 selectively bonds to S1, which would be related to the fact that the Cu site maintains a direct-above–direct-below relationship with the adjacent layer even in the 180° rotated layer as shown in Fig. 3 ▸.

## 4. Conclusions

We synthesized CuVP_2_S_6_ crystals and investigated its crystal structure. By selected-area electron diffraction and high-resolution scanning transmission electron microscopy, we determined that the space group of CuVP_2_S_6_ is unambiguously *C*2. We found that CuVP_2_S_6_ contains 180° rotation microtwins at the single-layer level. As a result of single-crystal X-ray structure analysis, we confirmed that characteristic octahedral rotations and the Cu site can be represented by a split atom model into three sites. The Cu3 atoms, which are at the least occupied site of the three sites and protrude into the van der Waals gap, were directly observed by high-resolution STEM. It was suggested that these Cu3 atoms provide additional (ionic) bonds locally to the van der Waals bonds between layers in the Cu*M*′′P_2_S_6_ system. Structural phase transitions associated with the electric phase transitions to the ferroelectric and antiferroelectric phases have been observed in CuInP_2_S_6_ (Maisonneuve *et al.*, 1997 ▸) and CuCrP_2_S_6_ (Susner *et al.*, 2020 ▸; Cho *et al.*, 2022 ▸), respectively. We believe that CuVP_2_S_6_ also exhibits a low-temperature structural phase transition and we are currently conducting research into this, which will be published elsewhere.

## Supplementary Material

Crystal structure: contains datablock(s) global, I. DOI: 10.1107/S2052520625008820/je5061sup1.cif

Structure factors: contains datablock(s) I. DOI: 10.1107/S2052520625008820/je5061Isup2.hkl

CCDC reference: 2494459

## Figures and Tables

**Table 1:** Crystal data and experimental details

Crystal data	
Chemical formula	CuP_2_S_6_V
*M*_r_	368.8
Crystal system, space group	Monoclinic, *C*2
Temperature (K)	293
*a*, *b*, *c* (Å)	5.9502 (3), 10.3126 (5), 6.6985 (3)
β (°)	107.221 (2)
*V* (Å^3^)	392.61 (3)
*Z*	2
Radiation type	Mo *K*α
μ (mm^−1^)	5.80
Crystal size (mm)	0.04 × 0.04 × 0.01
Crystal color	Black

Data collection	
Diffractometer	Bruker CCD
Absorption correction	Multi-scan (*SADABS*)
*T*_min_, *T*_max_	0.672, 0.746
No. of measured, independent and observed [*I* > 3σ(*I*)] reflections	1629, 640, 626
*R*_int_	0.032
(sin θ/λ)_max_ (Å^−1^)	0.714

Refinement	
*R*[*F* > 3σ(*F*)], *wR*(*F*), *S*	0.035, 0.141, 1.69
No. of reflections	640
No. of parameters	60
Δρ_max_, Δρ_min_ (e Å^−3^)	0.63, −0.70

**Table:** 

Site	Wyckoff position	*g*	*x*	*y*	*z*	*U*_eq_ (Å^2^)
Cu1	2*a*	0.293 (5)	0	0†	0	0.0535 (14)
Cu2	4*c*	0.262 (3)	0.0673 (9)	0.0061 (10)	0.2034 (13)	0.0535
Cu3	4*c*	0.091	0.114 (2)	0.007 (2)	0.345 (3)	0.0535
V1	2*a*	0.938 (3)	0	0.3437 (8)	0	0.0149 (4)
V2	2*a*	0.062	0	0.661 (3)	0	0.0149
P1	4*c*	0.938	0.0562 (2)	0.6754 (9)	0.1695 (2)	0.0094 (4)
P2	4*c*	0.062	0.067 (3)	0.335 (3)	0.172 (3)	0.0094
S1	4*c*	1	0.2316 (3)	0.5049 (10)	0.2524 (2)	0.0165 (4)
S2	4*c*	1	0.2513 (3)	0.1868 (10)	0.2482 (2)	0.0167 (7)
S3	4*c*	1	0.2657 (2)	0.8346 (9)	0.2487 (2)	0.0149 (5)

**Table:** 

Site	*U*_11_	*U*_22_	*U*_33_	*U*_12_	*U*_13_	*U*_23_
Cu1	0.041 (2)	0.027 (2)	0.097 (3)	0	0.027 (2)	0
Cu2	0.041	0.027	0.097	0	0.027	0
Cu3	0.041	0.027	0.097	0	0.027	0
V1	0.0157 (6)	0.0156 (7)	0.0130 (6)	0	0.0036 (4)	0
V2	0.0157	0.0156	0.0130	0	0.0036	0
P1	0.0076 (6)	0.0063 (6)	0.0147 (7)	−0.0016 (4)	0.0040 (4)	−0.0005 (5)
P2	0.0076	0.0063	0.0147	−0.0016	0.0040	−0.0005
S1	0.0176 (7)	0.0093 (7)	0.0187 (6)	−0.0028 (7)	−0.0004 (5)	0.0004 (6)
S2	0.0147 (7)	0.020 (2)	0.0174 (7)	0.0020 (5)	0.0085 (5)	0.0062 (5)
S3	0.0145 (6)	0.0139 (11)	0.0193 (7)	−0.0074 (7)	0.0094 (5)	−0.0054 (5)

† The coordinate is fixed to define the origin along *y* in space group *C*2.

**Table 3:** Selected interatomic distances (Å) in CuVP_2_S_6_

Cu1–S1	2.648 (2)	(×2)		V1—S1	2.478 (9)	(×2)
Cu1–S2	2.691 (7)	(×2)		V1—S2	2.479 (8)	(×2)
Cu1–S3	2.573 (6)	(×2)		V1—S3	2.470 (2)	(×2)
						
Cu2—S1	2.118 (6)			P1—S1	2.036 (12)	
Cu2—S2	2.137 (13)			P1—S2	2.037 (2)	
Cu2—S3	2.098 (12)			P1—S3	2.035 (11)	
						
Cu3—S1(intralayer)	2.175 (14)			P1—P1	2.169 (2)	
Cu3–S1(across vdW gap)	2.58 (2)					
Cu3—S2	2.20 (2)					
Cu3—S3	2.18 (2)					
